# Detection of zoonotic bacteria in wild Australian freshwater turtles

**DOI:** 10.1111/avj.70129

**Published:** 2026-08-06

**Authors:** TF Scheelings, TTH Van, RJ Moore, LF Skerratt

**Affiliations:** ^1^ Melbourne Veterinary School, Faculty of Science The University of Melbourne Werribee Victoria 3030 Australia; ^2^ School of Science RMIT University, Bundoora West Campus Bundoora Victoria 3083 Australia

**Keywords:** freshwater turtles, microbiota, pathogens, public health, wildlife health, zoonoses

## Abstract

Freshwater turtles are long‐lived, abundant components of Australian aquatic ecosystems, yet little is known about the diversity and prevalence of zoonotic bacteria they may carry. In this study, we conducted one of the most comprehensive surveys to date of zoonotic microorganisms associated with wild Australian freshwater turtles. A total of 257 individuals representing 10 species across 6 genera were sampled from multiple riverine and wetland systems. Using next generation sequencing, we detected 18 confirmed zoonotic bacterial species spanning 10 genera, including *Acinetobacter*, *Vibrio*, *Plesiomonas*, *Campylobacter*, and *Clostridium*. Nearly half of all turtles (49.8%) were shedding at least one zoonotic taxon at the time of capture, with some individuals carrying multiple species simultaneously. Several additional isolates from genera with known zoonotic potential could not be resolved to species level, highlighting gaps in current reference databases and the need for improved genomic characterisation. No *Salmonella* spp. were detected, consistent with previous research suggesting a low risk of reptile‐associated salmonellosis from Australian freshwater turtles. Shedding prevalence varied markedly among turtle species and locations, suggesting that host ecology and environmental conditions influence bacterial acquisition and persistence. Due to data sparsity for many taxa, analyses were limited to descriptive assessments. Overall, these findings demonstrate that Australian freshwater turtles harbour a diverse assemblage of bacteria with zoonotic relevance and may act as reservoirs within aquatic environments. Continued surveillance, integration of genomic tools, and consideration of human–wildlife interaction contexts, including cultural harvesting, will be important for assessing public health implications and informing conservation and biosecurity strategies.

Freshwater turtles are integral components of aquatic ecosystems, contributing to nutrient cycling, scavenging, and seed dispersal.^1^ Australia harbours a diverse assemblage of native freshwater turtle species, many of which are long‐lived and occupy a wide range of ecological niches, from tropical rivers to arid‐zone billabongs.^2^ As ectothermic, semi‐aquatic vertebrates with close contact with sediment, water, and other wildlife, turtles host complex microbial communities on their skin,^3^ shell,^4^ and within their gastrointestinal tracts.^5^ These microbial assemblages play important roles in host health, influencing digestion, immune function, and physiology.^6^

Recently high‐throughput sequencing has revealed that the gut microbiota of chelonians is shaped by multiple factors including diet,^7^ and habitat type.^8^ Furthermore, extrinsic factors such as water quality also drive variation in bacterial composition across populations and regions.^9^ Despite these insights, most microbiota studies in Australian turtles have focused on ecological or conservation aspects, with limited attention to the potential for pathogenic or zoonotic bacteria within these communities.

Globally, chelonians have been recognised as reservoirs for important zoonotic bacteria, most notably *Salmonella*,^10^ which has been linked to significant human‐health outbreaks, some with severe life‐threatening illness.^11^, ^12^, ^13^, ^14^, ^15^ In addition to *Salmonella*, chelonians have been reported to harbour other pathogens of public health significance including *Pseudomonas* spp., *Aeromonas* spp., and *Vibrio* spp.^16^ In Australia, reports of zoonotic bacteria in wild turtles are scarce and have been poorly investigated.^17^ Given the increasing frequency of human–turtle interactions through wildlife rescue, rehabilitation, and research activities, there is a pressing need to better characterise the occurrence and diversity of zoonotic bacteria in native turtle populations.

In this study, the bacterial communities associated with Australian freshwater turtles were investigated, with a focus on identifying taxa of zoonotic concern using 16S rRNA gene sequencing technology. This work provides a foundation for understanding the role of turtles as reservoirs of zoonotic microbes in Australian aquatic systems and contributes to improved biosecurity, public health, and wildlife management practices.

## Methods

### Study populations

Turtles were trapped and sampled under permit 10,010,480 from the Department of Environment, Land, Water and Planning (VIC), and permit number RP1497 from the Victorian Fisheries Authority (VIC), permit SL102774 from the Department of Climate Change, Energy, the Environment and Water (NSW), permit TFA 2324–0037 from the Department of Biodiversity, Conservation and Attractions (WA), permit number 266061 from the Department of Agriculture and Fisheries (QLD), and permit number WA0048559 from the Department of Environment and Science (QLD). Turtles were captured from multiple sites in four Australian states (Table 1) and all turtles were released alive at their point of capture immediately after sampling had been completed.

**Table 1:** Zoonotic bacterial species detected in wild Australian freshwater turtles.

Genus	Species	Total sampled	Location	Zoonotic bacteria	No (%) positive
*Chelodina*					
	Broad‐shell turtle (*Chelodina expansa*)	11	Moloney wetland (VIC, *n* = 8) Mary River (QLD, *n* = 3)	*Plesiomonas shigelloides*	2 (25)
				*Acinetobacter lwoffii*	1 (12.5)
				*Plesiomonas shigelloides*	1 (12.5)
				*Campylobacter blaseri*	1 (33.3)
				*Clostridium perfringens*	1 (33.3)
				*Acinetobacter lwoffii*	2 (66.7)
	Eastern long‐neck turtle (*Chelodina longicollis*)	78	Baranduda (VIC, *n* = 20) Barnawartha (VIC, *n* = 18) Darebin wetlands (VIC, *n* = 40)	*Plesiomonas shigelloides*	4 (20)
				*Plesiomonas shigelloides*	2 (10)
				*Acinetobacter lwoffii*	1 (5.6)
				*Aeromonas popoffii*	1 (5.6)
				*Plesiomonas shigelloides*	5 (12.5)
				*Vibrio metoecus*	1 (2.5)
				*Vibrio cholerae*	12 (30)
				*Pseudomonas otitidis*	9 (22.5)
				*Pasteurella testudinis*	10 (25)
	Oblong turtle (*Chelodina oblonga*)	20	Lake Claremont (WA, *n* = 10) Baigup wetlands (WA, *n* = 10)	*Plesiomonas shigelloides*	6 (60)
				*Vibrio cholerae*	4 (40)
				*Vibrio vulnificus*	1 (10)
				*Plesiomonas shigelloides*	4 (40)
				*Pasteurella testudines*	1 (10)
				*Clostridium perfringens*	1 (10)
				*Acinetobacter lwoffii*	2 (20)
*Elseya*					
	White‐throated snapping turtle (*Elseya albagula*)	21	Fitzroy River (QLD, *n* = 7) Mary River (QLD, *n* = 14)	*Acinetobacter guillouiae*	1 (14.3)
				*Acinetobacter schindleri*	1 (7.1)
				*Acinetobacter lwoffii*	11 (78.6)
*Elusor*					
	Mary River turtle (*Elusor macrurus*)	8	Mary River (QLD, *n* = 8)	*Staphylococcus pasteuri*	1 (12.5)
				*Clostridium perfringens*	1 (12.5)
				*Acinetobacter lwoffii*	5 (62.5)
*Emydura*					
	Macquarie River turtle (*Emydura macquarii*)	48	Bellinger River (NSW, *n* = 7) Moloney wetland (VIC, *n* = 9) Baranduda (VIC, *n* = 6) Fitzroy River (QLD, *n* = 10) Mary River (QLD, *n* = 16)	*Plesiomonas shigelloides*	1 (14.3)
				*Staphylococcus pasteuri*	1 (14.3)
				*Corynebacterium kroppenstedtii*	1 (14.3)
				*Staphylococcus pasteuri*	1 (11.1)
				*Pseudomonas otitidis*	3 (33.3)
				*Campylobacter blaseri*	3 (50)
				*Clostridium perfringens*	3 (30)
				*Acinetobacter guillouiae*	5 (50)
				*Acinetobacter schindleri*	1 (10)
				*Acinetobacter lwoffii*	11 (68.8)
*Rheodytes*					
	Fitzroy River turtle (*Rheodytes leukops*)	8	Fitzroy River (QLD, *n* = 8)	*Plesiomonas shigelloides* *Pseudomonas otitidis* *Acinetobacter baumannii* *Acinetobacter schindleri* *Acinetobacter guillouiae* *Acinetobacter variabilis* *Acinetobacter lwoffii* *Acinetobacter radioresistens*	2 (25) 1 (12.5) 3 (37.5) 4 (50) 5 (62.5) 1 (12.5) 4 (50) 1 (12.5)
*Myuchelys*					
	Western saw‐shelled turtle (*Myuchelys bellii*)	17	Kentucky reservoir (NSW, *n* = 17)	*Plesiomonas shigelloides*	2 (11.8)
				*Vibrio cholerae*	4 (23.5)
				*Staphylococcus pasteuri*	2 (11.8)
				*Acinetobacter lwoffii*	5 (29.4)
				*Acinetobacter radioresistens*	1 (5.9)
	Bellinger River turtle (*Myuchelys georgesi*)	32	Bellinger River (NSW, *n* = 32)	*Plesiomonas shigelloides*	2 (6.3)
				*Campylobacter blaseri*	10 (31.3)
				*Campylobacter insulaenigrae*	1 (3.1)
				*Staphylococcus pasteuri*	1 (3.1)
				*Clostridium perfringens*	6 (18.8)
				*Acinetobacter lwoffii*	1 (3.1)
	Saw‐shelled turtle (*Myuchelys latisternum*)	14	Mary River (QLD, *n* = 14)	*Plesiomonas shigelloides*	1 (7.1)
				*Clostridium perfringens*	2 (14.3)
				*Acinetobacter schindleri*	2 (14.3)
				*Acinetobacter lwoffii*	9 (64.2)

*Note:* The table summarises the number of individuals sampled per species, the geographic distribution of sampling sites, the zoonotic bacteria identified, and the number (percentage) of turtles shedding each taxon.

Abbreviations: NSW = New South Wales; QLD = Queensland; VIC = Victoria; WA = Western Australia.

### Sample collection

At all sites, turtles were captured using a variety of methods including baited and unbaited fyke nets, baited cathedral nets, modified funnel traps or by hand capture while snorkelling. The capture method used at each location was selected based on the behavioural characteristics of the target species and the experience of the field team. Following capture, each turtle was placed in dorsal recumbency, and a sterile cotton swab was inserted into the cloaca and twirled so that it contacted the cloacal mucosa. The swab was then retracted, and the tip cut using flame‐sterilised wire cutters and stored in 1 ml of ZymoBIOMICS DNA/RNA Shield (Integrated Sciences) in a sterile Eppendorf tube. The Eppendorf tubes were then stored in a portable ice box before being transported to the laboratory and stored at −80°C until DNA extraction could occur. To prevent the spread of pathogens to humans and between sites, turtles were handled with disposable gloves, and all equipment was cleaned, dried and sterilised between locations.

### DNA extraction

DNA was extracted using the ZymoBIOMICS DNA Miniprep Kit (Integrated Sciences) according to the manufacturer's instructions. Following extraction, DNA was stored at −80°C until amplicon sequencing could take place. For all turtles, the level of handling was kept to a minimum and they were released, unharmed, at their point of capture.

### 16S rRNA gene amplicon sequencing

The V3‐V4 region of 16S rRNA genes was polymerase chain reaction (PCR) amplified with forward primer 5′ ACTCCTACGGGAGGCAGCAG 3′ and reverse primer 5′ GGACTACHVGGGTWTCTAAT 3′ using Q5 high fidelity polymerase (New England Biolabs) with a dual barcoding strategy.^18^ The PCR cycling parameters were 98°C for 1 min, 35 cycles of 98°C for 10 s, 49°C for 30 s, and 72°C for 30 s, followed by a 10‐minute extension at 72°C. Sequencing was performed on an Illumina MiSeq system (2 × 300 bp).

### Data processing

Sequence data was analysed using Quantitative Insights into Microbial Ecology 2 (QIIME2) version 220.6,^19^ using the Divisive Amplicon Denoising Algorithm (DADA2) plugin for quality filtering, denoising, chimaera detection, and amplicon sequence variant (ASV) calling.^20^. Trimming was performed in DADA2 using the default parameters, and ASV's with fewer than 10 sequence reads across the entire data set were removed. ASVs were taxonomically classified using the SILVA database (v138.1),^21^ and an ASV abundance table with taxonomic assignments was produced for further analysis.

### Data analysis

Data was filtered and analysed in R (R Core Team 2024), utilising the package “phyloseq”.^22^ The *phyloseq* object, containing the ASV table, taxonomy assignments, and sample metadata, was used to explore the taxonomic composition of turtle‐associated microbiota. The taxonomy matrix was queried to detect genera and species previously recognised as zoonotic using functions such as tax_table() and logical filtering with subset_taxa(). For example, taxa matching known zoonotic genera (e.g., *Salmonella*, *Aeromonas*, *Mycobacterium*, *Pseudomonas*, and *Escherichia*) were extracted for further inspection. This workflow provided a reproducible framework to screen for bacterial taxa of zoonotic concern directly within the phyloseq taxonomy matrix, integrating microbial community profiling with pathogen surveillance. To visualise the presence/absence of zoonotic bacteria species in the data set a correlation matrix was created (Figure 1).

**Figure 1:**
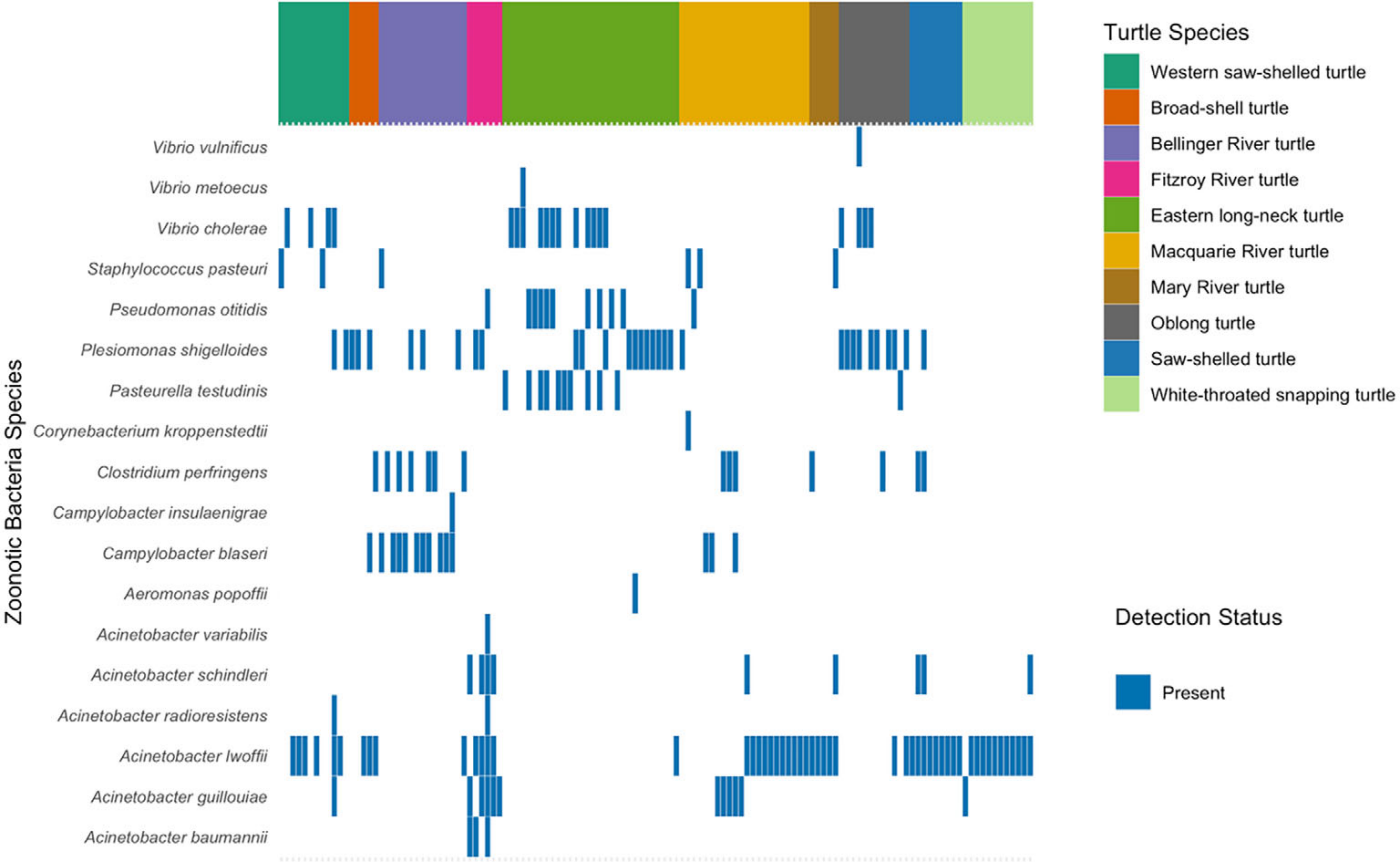
Schematic representation of the presence of zoonotic bacterial taxa detected across individual freshwater turtle samples. Each column represents a sample, and each row corresponds to a bacterial species, with blue tiles indicating detection. The coloured annotation bar above the heatmap identifies the turtle species associated with each sample, with distinct colours assigned to each species for clarity. Legends to the right indicate turtle species and bacterial detection status. This visualisation highlights variation in zoonotic bacterial profiles among species and across samples.

## Results

In total, 257 turtles were captured comprising 10 species representing 6 genera (Table 1). Of these turtles 128 (49.8%) individuals were shedding a least one zoonotic bacterium at the time of sampling (Mean 1.5 ± 0.9). One turtle, a Fitzroy River turtle, tested positive for seven different zoonotic species. We identified 18 species of known zoonotic bacteria, belonging to 10 genera (Table 1). This was comp of six species of *Acintobacter*, three species of *Vibrio*, two species of *Campylobacter*, and one species from each of the genera *Aeromonas*, *Clostridium*, *Corynebacterium*, *Pasturella*, *Plesiomonas*, *Pseudomonas*, and *Staphylococcus* (Table 1). In addition, bacteria from a further six genera with zoonotic potential were isolated including *Citrobacter*, *Enterobacter*, *Serratia, Chromobacterium*, *Neisseria* and *Klebsiella* but these could not be identified to a species level. The most common species identified was *Acintobacter lwoffii* (*n* = 53), followed by *Plesimonas shigelloides* (*n* = 32), *Vibrio cholerae* (*n* = 20), *Campylobacter blaseri* (*n* = 14) and *Clostridium perfringens* (*n* = 14).

The turtle species most frequently shedding zoonotic bacteria was the Fitzroy River turtle (*n* = 6, 75%), followed by the western saw‐shelled turtle (*n* = 11, 64.7%), saw‐shelled turtle (*n* = 9, 64.3%), Mary River turtle (*n* = 5, 62.5%), oblong turtle (*n* = 12, 60%), white‐throated snapping turtle (12, *n* = 57.1%), Bellinger River turtle (*n* = 15, 46.9%), Macquarie River turtle (*n* = 22, 45.8%), Broadshell turtle (*n* = 5, 45.5%) and the eastern long‐neck turtle (*n* = 30, 38.5%). The location with the highest number of turtles shedding zoonotic bacteria was Kentucky Reservoir (*n* = 12, 70.6%), followed by Lake Claremont (*n* = 7, 70%), Mary River (*n* = 38, 69.1%), Darebin Wetlands (*n* = 23, 57.5%), Fitzroy River (*n* = 14, 56%), Baigup Wetlands (*n* = 5, 50%), Bellinger River (*n* = 17, 43.6%), Moloney Wetland (*n* = 5, 29.4%), Barnawartha (*n* = 3, 16.7%), and Baranduda (*n* = 4, 15.4%). Although 257 turtles were sampled, the sparsity of detections across samples meant that the dataset lacked sufficient replication and coverage for reliable differential abundance testing or more advanced statistical analyses. As a result, only descriptive assessments were conducted.

## Discussion

This study provides one of the most comprehensive assessments to date of zoonotic bacteria associated with Australian freshwater turtles, revealing that nearly half (49.8%) of sampled individuals were shedding at least one zoonotic taxon at the time of capture. The detection of 18 confirmed zoonotic bacterial species across 10 genera demonstrates that native turtles harbour a diverse assemblage of microorganisms with potential relevance for both animal and human health. While turtles are known reservoirs for a range of pathogens internationally, the broader suite of zoonotic taxa identified here, including *Acinetobacter*, *Vibrio*, *Plesiomonas*, *Campylobacter*, and *Clostridium*, indicates a more complex zoonotic microbial landscape within Australian turtle populations than previously recognised.

The high prevalence of *Acinetobacter* species aligns with their known ubiquity in aquatic environments and their association with opportunistic infections in humans and animals.^23^, ^24^, ^25^ This genus is of particular concern as an emerging zoonosis as some species, including *A. baumannii*, overexpress intrinsic β‐lactamases and multidrug resistant (MDR) genes.^26^, ^27^ MDR *Acinetobacter* spp. have been isolated from a wide range of chelonian species globally,^28^, ^29^, ^30^ indicating the importance of these reptiles as reservoirs for this genus. Similarly, the species *Plesiomonas shigelloides* frequently inhabits freshwater ecosystems where it can be bioaccumulated in vertebrate and invertebrate hosts.^31^ *Plesiomonas shigelloides* infections from pet turtles have been implicated in sepsis and near death in human patients.^32^ *Vibrio* spp., in particular *V. cholerae*, are frequently isolated from a range of marine turtle species,^33^, ^34^ where they have been identified as important zoonoses in communities that rely on sea turtle products for subsistence living.^35^ They have also been isolated from pet turtles where they are a source of significant zoonotic risk for reptile keepers.^36^ The identification of other zoonotic bacteria such as *Campylobacter blaseri* and *Clostridium perfringens* further highlights the capacity for turtles to carry pathogens associated with gastrointestinal disease in humans.^37^, ^38^ Importantly, a single Fitzroy River turtle was found to be shedding seven zoonotic species, demonstrating that individual turtles can act as multi‐pathogen reservoirs and potentially play an outsized role in environmental dissemination.

Interestingly, we did not identify any *Salmonella* spp. in turtles in this investigation. This result is consistent with previous explorations into the prevalence of *Salmonella* in Australian reptiles in which this bacteria was rarely found in freshwater turtles.^17^ Combined, the results of these studies indicate that Australian freshwater turtles are unlikely to be a source of reptile‐associated salmonellosis in humans. Conversely, chelonians continue to pose a significant public health concern for human salmonellosis in most other parts of the world,^10^, ^39^ and significant effort is directed toward monitoring and managing this risk. The apparent absence of *Salmonella* in Australian turtle populations therefore represents an important point of divergence and highlights the need to better understand the ecological, environmental, and evolutionary factors that underlie this distinction.

In addition to the broader public health considerations, these findings have relevance for Indigenous communities in northern Australia where freshwater turtles continue to hold nutritional, cultural, and ceremonial importance.^40^ Although traditional preparation methods may reduce some microbial risks, the presence of zoonotic bacteria raises important questions regarding potential exposure pathways for people who harvest, butcher, or consume turtles. While there are currently no documented cases of turtle‐associated zoonoses within Australian Indigenous populations, the diversity of bacteria identified in this study highlights the need for targeted health communication, co‐developed with Traditional Owners, that respects cultural practices while supporting safe handling and food preparation.

Shedding prevalence varied substantially among taxa and turtle species, suggesting species‐specific susceptibility, behaviour, or habitat preferences that may influence bacterial acquisition and persistence. The Fitzroy River turtle, western saw‐shelled turtle, and saw‐shelled turtle had the highest proportions of individuals shedding zoonotic bacteria. These species typically inhabit riverine or slow‐flowing systems with high organic loads, which may facilitate bacterial proliferation. Likewise, shedding frequency differed among sampling locations, with the highest proportions observed in Kentucky Reservoir, Lake Claremont, and the Mary River. Such patterns may reflect environmental drivers such as climate change,^41^ water quality,^42^ and anthropogenic disturbance,^43^ that influence bacterial abundance and transmission dynamics.

Although this study provides important baseline data, several limitations must be acknowledged. Due to the sparsity of detections for many taxa, the dataset lacked the replication required to conduct robust differential abundance testing or more complex statistical analyses. As a result, our interpretations are based on descriptive trends rather than inferential comparisons. Additionally, several isolates could not be identified to species level despite belonging to genera with known zoonotic potential, underscoring the need for further work incorporating whole‐genome sequencing or improved culture‐independent methods to more accurately resolve pathogen diversity. Importantly, future investigations should include screening for antimicrobial resistance determinants, as the presence of multidrug‐resistant genes in turtle‐associated bacteria would have significant implications for both wildlife and public health.

The findings of this investigation have clear implications for wildlife management, public health, and rehabilitation practices. Many of the zoonotic bacteria identified here are opportunistic pathogens capable of causing disease in humans, particularly those who handle turtles regularly, such as wildlife carers, researchers, and veterinarians. While the overall risk of transmission remains uncertain, the high prevalence of shedding in some species and locations suggests that appropriate hygiene measures and risk mitigation strategies should be standard practice. From an ecological perspective, freshwater turtles may contribute to the maintenance and movement of bacterial pathogens within aquatic systems, although further research is needed to quantify their epidemiological significance relative to other wildlife and environmental reservoirs.

Ultimately, this study highlights the importance of continued monitoring of zoonotic bacteria in freshwater turtle populations across Australia. Future work incorporating longitudinal sampling, quantitative microbial load assessments, and environmental context will be essential to understanding the drivers of pathogen occurrence and the potential consequences for both turtles and humans. The diverse range of zoonotic taxa detected here underscores the value of turtles as sentinels of aquatic microbial health and provides a foundation for improved biosecurity and conservation planning.

## Conflicts of interest and sources of funding

The authors declare no conflicts of interest. T.F.S. was supported by a McKenzie Postdoctoral Fellowship from the University of Melbourne.

## Ethics statement

This study was approved by The University of Melbourne Office of Research Ethics and Integrity (Ethics ID: 2022‐24808‐32226‐4) and all experiments were performed in accordance with relevant guidelines and regulations.

## Data Availability

The datasets generated and/or analysed during the current study are available in the *The National Center for Biotechnology Information* www.ncbi.nlm.nih.gov and may be made available on reasonable request from the authors.
